# Meat Nutritional Value and Exoskeleton Valorisation of *Callinectes sapidus* from Three Sites of Biological and Ecological Interest in Morocco: Scientific Insights Toward a Management Strategy in the Mediterranean Sea

**DOI:** 10.3390/md23090367

**Published:** 2025-09-21

**Authors:** Kamal Gourari, Youness Mabrouki, Abdelkhaleq Fouzi Taybi, Abdessadek Essadek, Valentina Tanduo, Fabio Crocetta, Ilyesse Rahhou, Chaouki Belbachir, Lucia Rizzo, Bouchra Legssyer

**Affiliations:** 1 Laboratory for the Improvement of Agricultural Production, Faculty of Sciences, Biotechnologies and the Environment, Mohamed Premier University, Oujda 60000, Morocco; 2 Laboratory of Biotechnology, Faculty of Sciences of Dhar El Mehraz, Conservation and Valorization of Bioresources, Sidi Mohamed Ben Abdellah University, Fez 30003, Morocco; 3 Équipe de Recherche en Biologie et Biotechnologie Appliquées, Faculté Pluridisciplinaire de Nador, Université Mohammed Premier, Selouane, Nador 62700, Morocco; 4 Laboratory of Bioresources, Biotechnology, Ethnopharmacology and Health, Biochemistry Applied to the Valorization of Bioresources, Mohammed First University, Oujda 60000, Morocco; 5 Stazione Zoologica Anton Dohrn, Department of Integrative Marine Ecology, Villa Comunale, 80121 Napoli, Italy; fabio.crocetta@szn.it (F.C.); 6 Higher Institute of Nursing Professions and Health Techniques of Oujda (ISPITSO), Oujda 60000, Morocco; 7 Institute of Sciences of Food Production, National Research Council (CNR-ISPA), Via Lecce Monteroni, 73100 Lecce, Italy; 8 National Interuniversity Consortium for Marine Sciences (CoNISMa), Piazzale Flaminio 9, 00196 Rome, Italy

**Keywords:** alien species management, biological invasions, blue crabs, circular economy, chitin, chitosan, phenotypic plasticity

## Abstract

Biological invasions threaten biodiversity worldwide. The American blue crab *Callinectes sapidus* Rathbun, 1896, among the Mediterranean’s most damaging invaders, takes up the challenge to transform this threat into gain. To turn its impact into economic value and guide control efforts, we analysed separately the meat composition and exoskeleton biopolymers of adult crabs from three Moroccan protected Sites of Biological and Ecological Interest: Marchica Lagoon (S_1_), Moulouya Estuary (S_2_), and Al Hoceima National Park (S_3_). Marchica specimens exhibited the highest protein content (21.87 ± 1.15 g 100 g^−1^, *p* < 0.001) and an elevated lipid fraction, yielding nutrient-dense meat suitable for premium markets. Moulouya crabs were noted for their taste potential, with a higher concentration of fat (1.73 ± 0.09%) and carbohydrates (0.91 ± 0.1%). Al Hoceima individuals displayed markedly mineralised exoskeletons producing lean and low-fat meat, valued in dietary applications. Exoskeleton organic-to-mineral (OM/MM) ratios and proximate composition revealed three adaptive profiles, opportunistic (S_1_), acclimatory (S_2_), and conservative (S_3_), presumably correlated to local salinity, productivity, and substrate conditions, underscoring the species’ phenotypic plasticity. X-ray diffraction confirmed the α-chitin polymorph, while FTIR analysis indicated degrees of deacetylation consistent with high-purity chitosan. These findings support the development of a site-specific circular economy framework and may contribute to the ecological resilience of Morocco’s protected coastal areas.

## 1. Introduction

Biological invasions are a major threat to biodiversity and ecosystem functioning worldwide, and this phenomenon already affects almost all ecosystems, including aquatic environments [1]. The Mediterranean region is one of the renowned hotspots for non-indigenous species introduction and spread [2,3], with wetlands (estuaries, lagoons, and deltas) often hosting the highest number of non-indigenous species [4,5]. This is particularly nocuous, as wetlands not only act as a natural transition between terrestrial and aquatic ecosystems, thus somehow influencing both environments, but also are home to a wide range of global and regional biodiversity, providing and sustaining the basic economic and ecologic services for the local populations [6,7,8,9]. The same also holds true for Morocco, where several wetlands already designed as Sites of Biological and Ecological Interest (SBEIs) are characterised by high concentrations of rare or threatened endemic plant and/or animal species or are ecosystems with high biodiversity indexes [10]. However, in the last decades, these areas also faced the advent of biological invasions, with detrimental consequences on the native biota or on the local economy [11,12,13].

Among non-indigenous species affecting wetlands but also the wider coastal ecosystems, the American blue crab *Callinectes sapidus* Rathbun, 1896 (Crustacea: Decapoda: Portunidae) has now become a prominent species that in the recent decade has gained major attention from the media and scientists [14]. Adults are omnivorous predators, with a flexible diet that includes algae, molluscs, crustaceans, and small fish, thus impacting different trophic levels and phyla [15,16,17]. It is also an eurytherm species, thus tolerating a wide range of temperatures, and a euryhaline species, being able to adapt to a wide range of salinities [18]. The species also has a high reproductive rate and mates frequently [19,20]. Because the spread of *C. sapidus* coincides with declines in several species, including commercially important ones, by predator pressure and competition, it is considered as one of the worst invasive aquatic taxa in the Mediterranean Sea [21,22,23]. With regards Morocco, the species was first recorded on its coast in 2017 [22], several decades after the first reports in other Mediterranean countries [23]. Notwithstanding that, and in the complete absence of concrete actions to mitigate its invasion, in less than a decade *C. sapidus* has already spread all over the Mediterranean coastline and even reached the Atlantic side of Morocco, thus becoming a local pest [24,25,26].

Several management strategies have already been proposed or discussed to face this widespread and concomitant bioinvasion, with the final aim to downgrade the local population size and, at the same time, to turn a threat into an unprecedented scientific, technological, and economic opportunity [27,28,29,30,31]. Among them, not only the use of its meat as a major source of food, but also the use of the common “crab waste” to generate additional income for several peripheral communities [25,30,31]. In this context, decapods can be an important source of chitin (2-Acetamide-2-Deoxy-D-glucose) [32], whose deacetylated form, chitosan (2-amino-2-Deoxy-D-glucose and 2-Acetamide-2-Deoxy-2-D-glucose), may be used in several human domains. As an example, chitin extracts can potentially be used to prevent gastric ulcers induced by ethanol [33]. Thanks to its positive charges and its ability to modify the cell permeability of bacteria, chitosan also possesses antimicrobial activities and inhibits the growth of *Staphylococcus aureus* and *Escherichia coli* [34]. Furthermore, it is an antioxidant as it can trap free radicals and exhibit anticancer activities, thus being used for encapsulating anticancer agents by negatively targeting the production of tumour necrosis factors by macrophages [35]. In agriculture, chitosan can play an effective antifungal role for biological control [36]. In wastewater treatment, it is a good chelator in the flocculation phase [37]. Finally, thanks to the good film-forming properties of chitosan, it is also used in the field of food preservation and packaging to avoid the use of chemical preservatives [38].

In this context and in the view of its sustainable exploitation, the aim of this study was twofold: (i) to explore the meat yield of *C. sapidus* and its nutritional values in three different SBEIs located on the Mediterranean coast of Morocco, each of them being characterized by different environmental features; and (ii) to assess the exoskeleton extraction and the physicochemical characteristics (solubility, water content, and crystallinity) of its chitin and chitosan, comparing it with values obtained from shrimps, commonly used in the country for the same purpose. The present effort accounts for the first multidisciplinary project targeting non-indigenous species in such a way along the Mediterranean coast of Africa, tests the possibility of switching the exploitation strategy of *C. sapidus* in Morocco from a general blue bioeconomy (the sustainable application of life sciences and innovation to use and convert living aquatic resources into value-added products and services) toward a blue circular bioeconomy (an emerging economic model that uses byproducts and waste to create sustainable products and services within a closed-loop system), and contributes to the general knowledge of a non-indigenous species already widespread in the Mediterranean Sea. It finally tries to lower the impact of human pressures on the native biota and proposes a new exploitation model for this species in a yet underdeveloped country, potentially improving the future quality of life of the local communities.

## 2. Results

### 2.1. Morphological and Yield Characteristics of Callinectes Sapidus

Species identification was confirmed with published diagnostic characters, and only individuals with carapace width across the lateral spines > 10 cm and body mass > 110 g were retained (Table S1).

**Figure 1:**
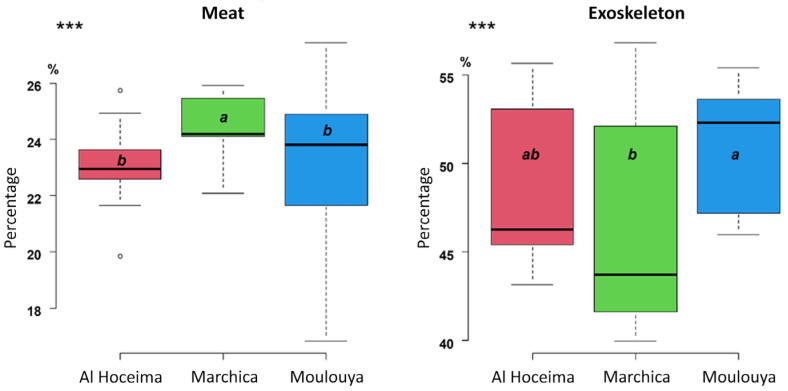
Meat and exoskeleton yields in *C. sapidus* from the three investigated Sites of Biological and Ecological Interest. *** indicates *p* < 0.001, different letters indicate significant differences between the investigated sites.

Meat and exoskeleton yields showed different results among sites (*n* = 33 per site, adults only). Meat yield differed among sites (one-way ANOVA: F(2.96) = 7.05, *p* = 0.00139) (Figure 1) and was higher at Marchica Lagoon (S_1_) (24–25%), intermediate at Moulouya (S_2_) (23–24%), and lower at Al Hoceima (S_3_) (22–23%). A one-way ANOVA followed by Tukey’s HSD showed that S_1_ crabs (group a) differed significantly (*p* < 0.001) from both S_2_ and S_3_ (group b), which were not different from each other. Exoskeleton yield also varied (F(2.96) = 7.92, *p* = 0.000654), with S_2_ showing the highest shell fraction (51.00 ± 3.22%; group a), S_1_ the lowest (46.62 ± 5.68%; group b), and S_3_ intermediate (49.14 ± 4.21%; group ab). All differences were significant (*p* < 0.001).

### 2.2. Physicochemical Characterization of Meat and Exoskeleton

The proximate composition of *C. sapidus* tissues across the three sites is summarized in Table 1 and Table S2, with both meat and exoskeleton fractions displaying clear spatial trends that mirror the three sampled environments.

#### 2.2.1. Meat

Blue crabs from S_1_ displayed a dry matter (DM) content of roughly 15.38 ± 0.49% of the wet weight. Organic matter (OM) accounted for 12.75 ± 0.45% of the dry weight, whereas inorganic matter (measured as ash) represented 2.89 ± 0.21% of the dry matter, being higher than values found in the other two sites (Table 1). The meat organic matter/mineral matter (OM/MM) ratio in the blue crab meat from S_1_ was the lowest recorded (4.41 ± 0.36). The meat of blue crabs collected from S_1_ contained the highest protein level (21.87 ± 1.15%), compared with 18.46 ± 1.07% and 17.11 ± 1.17% of S_2_ and S_3_, respectively, and slightly more lipids (1.98 ± 0.05%). Reducing sugars (0.56 ± 0.15%) were lower than the values found in S_2_ but higher compared to the percentages found in the samples from S_3_.

Crabs from S_2_ possessed an average dry matter content of 15.64 ± 2.85%, similar to S_1_. They exhibited the highest organic fraction (13.71 ± 1.70%) and the lowest inorganic residue (1.67 ± 0.58%). Consequently, the meat OM/MM ratio (organic matter/mineral matter) rose to 8.21 ± 1.03, signalling exceptionally mineral-poor meat. Proteins (18.46 ± 1.07%) were modestly below S_1_ values, whereas reducing sugars reached 0.91 ± 0.1%, being the highest among all sites, while lipids remained low (1.73 ± 0.09%).

Crabs from S_3_ exhibited the lowest dry matter content (13.49 ± 0.70%), implying a highly hydrated musculature. Organic matter (12.17 ± 0.12%) approximated S_1_ proportions, whereas inorganic matter was a mere (1.32 ± 0.06%). The meat OM/MM ratio (9.22 ± 0.43) was the highest, reflecting minimal mineral residues. Proteins (17.11 ± 1.17%) and lipids (1.56 ± 0.04%) were the lowest and reducing sugars (0.41 ± 0.09%) were similarly scant.

#### 2.2.2. Exoskeleton

The blue crab exoskeletons sampled from S_1_ contained 87.82 ± 1.35% of DM after desiccation (Table 1). They bore the greatest organic fraction of the three sites (37.43 ± 1.12% of dry weight, largely chitin and structural proteins) and thus the lowest inorganic fraction (48.52 ± 2.05%, measured as ash). Its OM/MM ratio (0.77 ± 0.048) far exceeded those of S_2_ and S_3_, denoting comparatively less mineralised exoskeletons.

S_2_ exoskeletons possessed the lowest dry matter fraction (75.4 ± 0.96%), a characteristic of less densely mineralised or recently moulted exoskeletons. Organic matter constituted only 26.89 ± 0.29%, the lowest proportion observed, whereas inorganic matter reached 50.39 ± 0.21%. The resulting OM/MM ratio (0.53 ± 0.007, second lowest) indicated a highly mineral exoskeleton relative to its organic framework.

The exoskeletons of blue crabs from S_3_ were the driest and the most mineralised: dry matter reached 93.91 ± 0.29%, organic matter 30.74 ± 0.47%, and inorganic matter 62.17 ± 0.15% of the DM, yielding an OM/MM ratio of 0.49 ± 0.008, the lowest among all sites.

### 2.3. Chemical Extraction and Characterization of Chitin and Chitosan

Chitin yields varied significantly among collection sites (Table 2). Processing 100 g of dried exoskeleton powder per batch produced average recoveries of 17.28% at S_1_, 12.23% at S_2_, and 10.30% at S_3_; the commercial reference sample (T) was not subjected to extraction and therefore has no comparable yield. The infrared (FTIR) spectral profile of the extracted chitosan matched previously published spectra, confirming the presence and purity of chitosan extracted from *C. sapidus* exoskeletons.

Normalised to whole-animal weight, the recoveries of chitosan represented 15.67%, 13.59%, and 14.50% of total crab mass, respectively, with a grand mean of 14.59 ± 1.04% chitosan per sampled individual. Consequently, S_1_ exoskeletons constituted the most attractive feedstock, delivering roughly one-fifth more polymer than S_2_ and nearly double that of S_3_.

#### 2.3.1. Physicochemical Characterization of Chitin

**Figure 2:**
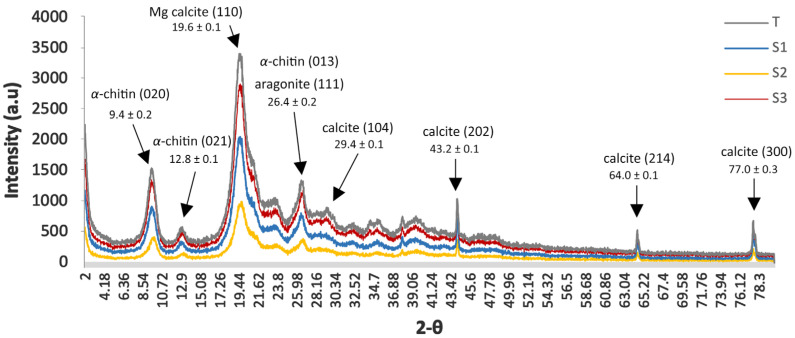
X-ray diffraction (XRD) of chitin extracted chemically from *C. sapidus* exoskeletons in the three SBEI areas. The vertical axis represents the intensity scale covering the full range of the XRD detector from 147 to 2489 counts.

The results of X-ray diffraction (XRD) (crystallinity index CrI and crystallite size D) are reported in Figure 2 and Table 3.

Samples S_1_ and S_2_ showed high crystallinity (84.42%, Table 3) coupled with an anisotropic growth in S_1_ and S_2_ that exhibited the largest crystallites on the densely packed (120) plane (6.6 and 7.1 nm, respectively). S_3_ exhibited a slightly lower CrI (80.95%). The untreated control T (the pink shrimp *Parapenaeus longirostris*) presented the least crystalline (74.1%) and T consistently recorded the smallest domains (3.5–6.7 nm), confirming its role as a low-crystallinity reference.

The crystallographic analysis uncovered a small but reproducible anisotropy for blue crab samples, as S_2_ was slightly more laterally coherent at the expense of the poorer vertical alignment of its microfibril body. These structural gradients may result in quantifiable differences in both mechanical stiffness and deacetylation kinetics—two properties of particular interest when designing chitosan extraction processes and downstream biotechnological applications.

**Figure 3:**
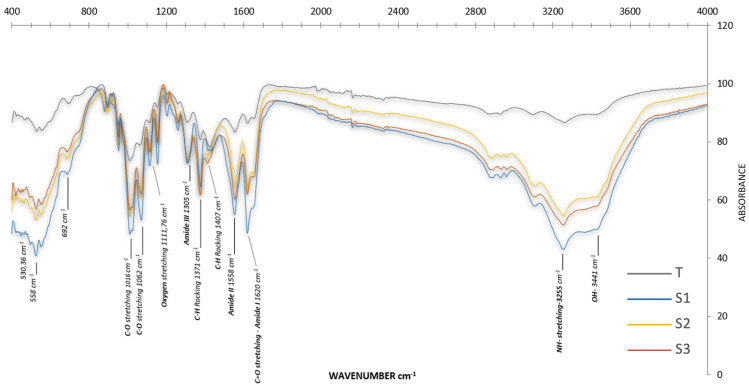
Spectra of percentage (%) transmittance of the FTIR spectrum of chitin extracted from *C. sapidus* exoskeletons S_1_, S_2_, S_3_, and control chitin (T).

The functional-group fingerprint and confirmation of the chitin structure are shown by the FTIR output. The FTIR spectra of α-chitin from the three sampling sites (Figure 3) showed differences mainly in the wave number bands, which indicated differences in the ecosystem-specific degree of acetylation (DA). A strong absorption peak at 1450 cm^−1^ in all studied areas confirmed that the extracted chitin was protein free, as noted by Alimi et al. [34]. The spectra revealed three characteristic amide bands corresponding to the vibrational modes of the CONH group: Amide I: 1619–1620 cm^−1^, Amide II: 1358 cm^−1^, and Amide III: 1305 cm^−1^. Intensive bands attributed to C-O-C and C-O stretching vibrations were observed at 530, 558, and 692 cm^−1^. In addition to methyl group bending bands seen between 1371 and 1407 cm^−1^, polysaccharide-specific vibrational bands were found between 525.51 and 1072.23 cm^−1^. The reference spectrum of chitin isolated from the pink shrimp (*Parapenaeus longirostris*; control T) was used to benchmark band positions and to validate the estimates of FTIR-based degree of acetylation (DA). The site-resolved assignments and the comparison with the shrimp reference (T) are summarised in Table S3.

#### 2.3.2. Physicochemical Characterization of Chitosan

**Figure 4:**
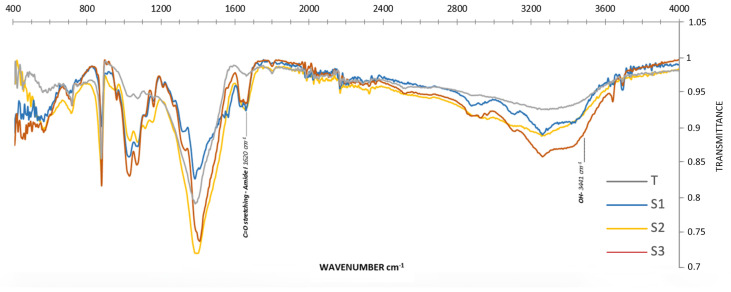
Spectra of percentage (%) transmittance of the FTIR spectrum of chitosan extracted from *C. sapidus* exoskeletons recorded at 4000–500 cm^−1^ chemically S_1_, S_2_, S_3_, and control chitosan (T).

The functional-group fingerprint and confirmation of the chitosan structure are shown in Figure 4 with assignments listed in Table 4. Important structural alterations brought by the deacetylation process are highlighted in the chitosan transmittance spectrum. In all chitosan samples, the broad O–H/N–H stretching envelope extended from ~3600 to 3000 cm^−1^, with an intensity maximum near ~3300–3350 cm^−1^; a lower-wavenumber shoulder around ~3117 cm^−1^ was evident in our spectra and reflected strong intra/intermolecular hydrogen bonding. Relative to α-chitin, the amide I (~1655–1620 cm^−1^) and amide II (~1560–1540 cm^−1^) bands were markedly attenuated but not absent, consistent with residual N-acetyl groups at DDA ≈ 79–84%. Concomitantly, the primary amine N–H bending appeared near ~1590–1600 cm^−1^ and grew in intensity with deacetylation. The C–H stretching region (ca. 2920–2870 cm^−1^) and polysaccharide C–O/C–O–C bands (~1150–1000 cm^−1^) were retained, with modest intensity changes after deacetylation. These features collectively confirmed successful deacetylation from α-chitin to chitosan while matching established chitosan spectral assignments [39]. The CH_2_ stretching near ~2930 cm^−1^ decreased in relative intensity in chitosan compared with the chitin control [40].

#### 2.3.3. Degree of Deacetylation (DDA) of Chitosan

The FTIR results showed that all field-extracted samples were highly deacetylated chitosan, whereas the commercial reference was modestly conversed (61.2%). Samples S_2_ and S_1_ gave the highest degree of deacetylation (DDA = 78.5–79.4%), followed by S_3_ (74.6%), as reflected by their low amide-band absorbance (A1620) relative to the hydroxyl reference (A3441) (Table 5).

## 3. Discussion

### 3.1. Physicochemical Characterization of Meat and Exoskeleton

The present study explored the meat and exoskeleton yield of *C. sapidus* along with the nutritional composition of its meat, across three different Sites of Biological and Ecological Interest (SBEIs) located on the Mediterranean coast of Morocco. Notable differences in composition and biochemical characterization across the three study sites—Marchica Lagoon, Moulouya Estuary, and Al Hoceima National Park—were observed, reflecting the influence of site-specific ecological pressures and the adaptive physiological strategies employed by *C. sapidus* in response to their respective environments. Indeed, these sites are characterized by different environmental features, as salinity regimes, food availability, and substrate qualities can shape crab physiology and, consequently, the proximate overall structure and the composition of tissues and exoskeleton. The findings indeed confirmed that the edible meat yield from the Marchica Lagoon was significantly higher than the values obtained from the two other populations. By contrast, the highest crab exoskeleton yield was found in the Moulouya Estuary, followed by Al Hoceima National Park, and finally Marchica Lagoon. This result aligns with the non-linear scale between meat and exoskeleton yields with size [41].

#### 3.1.1. Influence of the Ecological Context on the Biochemical Profile of the Meat

With regards biochemical composition of crab meat, *C. sapidus* specimens from the Marchica Lagoon are unequivocally nutrient dense, combining an elevated protein signature with a discernible retention of residual minerals. On average, the muscle tissue contains 21.87 g of protein per 100 g of dry matter (about 20% higher than crabs collected in the Moulouya Estuary and 28% higher than those from Al Hoceima National Park), and this protein accounts for roughly ~86% of the dry tissue’s organic matter. These values correspond to roughly 40% of the Food and Agriculture Organization of the United Nations (FAO) and World Health Organization (WHO) daily requirement for an average adult (~50 g) and just below the 20–25 g per-meal intake shown to maximise muscle–protein synthesis—highlighting its value as a lean and nutrient-dense food that benefits both ecological management and human nutrition [42,43,44]. Such values position the Marchica population as an exceptional natural source of high-quality crustacean protein. The nutrient profile of *C. sapidus* at Marchica Lagoon points to a strategy centred on anabolic tissue growth and energy storage. Crabs from the lagoon show a substantial lipid store in line with previous studies reporting crab meat rich in omega-3 fatty acids (EPA and DHA), unsaturated fatty acids, and other healthy elements [45,46,47].

This biochemical composition most plausibly reflects a diet rich in animal protein [48] as well as a greater ingestion—or at least adsorption—of fine mineral particles and dissolved salts that are continuously suspended in the lagoon environment, possibly influenced by fluvial sediment loads [49]. Indeed, the Marchica Lagoon is linked to the sea yet receives watershed inputs, including legacy iron mine discharges, and thus, this confined, nutrient-rich system supplies abundant prey (e.g., molluscs, organic detritus), fostering the high protein and lipid levels observed in the meat of individuals sampled from this site. The moderate (15.38%) dry matter is consistent with absolute values typically observed in crabs from lagoon ecosystems, where water retention tends to be lower than in fully marine habitats due to the osmotic stress caused by fluctuating salinity levels (22–39 PSU) [50]. Therefore, this balance between hydration and nutrient density increases the suitability of the meat for both fresh consumption and preservation (e.g., freezing, canning).

The Moulouya Estuary, where freshwater from the Moulouya River, the largest river in North Africa (Maghreb), mixes with Mediterranean seawater, represents a highly dynamic ecosystem. It is characterized by a marked salinity gradient (~30–35 PSU) [51], which imposes variable osmoregulatory requirements on resident species. This gradient is shaped by the influx of mineral-rich sediment originating from upstream agricultural and geological sources [52], as well as by nutrient pulses from seasonal flooding providing phytoplankton blooms and detrital biomass, that drive the basal compartment of the estuarine food web [53]. Indeed, specific minerals (e.g., iron, calcium) could play a crucial role in influencing crab tissue composition. These conditions determine the physiological and biochemical traits of *C. sapidus*, promoting adaptations that allow for the right trade-off between osmoregulation, calcification, and energy investment, resulting in a different biochemical profile [54]. Indeed, the meat of estuarine crabs contains markedly elevated levels of reducing sugars, a pattern that points to substantial glycogen stores and to hyperglycaemic responses induced by brackish water stress, knowing that reducing sugars (e.g., glucose, fructose) and glycogen (stored carbohydrate) are key energy substrates mobilized during fasting, physiological stress, or high activity to maintain metabolic demands. In particular, the high reducing sugar content likely reflects hypo-osmotic stress conditions that raise energetic costs of osmoregulation and induce hyperglycaemia in these organisms [55]. Such energetic demands may limit lipid storage, aligning with the lower lipid levels measured.

The bay of Al Hoceima National Park represents a markedly different marine environment, distinguished by ecological stability, balanced predator–prey dynamics, and characteristic metabolic responses. The rocky substrate in the bay, with seagrass meadows providing many microhabitats for preys (e.g., molluscs, small fish) [56,57] and a limited anthropogenic presence, promotes a good ecological status. These conditions might have effects on the physiology and diet of crabs, partially elucidating the biochemical pattern observed in this population [25,26]. The composition of crab meat from Al Hoceima shows a relatively low protein and low lipid content that suggests minimal energy reserves, an adaptation that matches the oligotrophic and crystal-clear waters of Al Hoceima National Park, where prey is sparse and energetically costly to obtain [58]. Conversely, crabs from more productive but unstable habitats (i.e., Moulouya Estuary, Marchica Lagoon) tend to store energy in muscle tissue, while those from preserved yet physically demanding ecosystems (i.e., Al Hoceima National Park) favour structural robustness. The crab meat from Al Hoceima also exhibits the lowest dry matter content, implying a highly hydrated musculature—a likely consequence of stable marine conditions or size/sex structure of the catch. The leaner muscle composition indicates that stored reserves are predominantly used for maintenance and survival, consistent with a habitat where food supply meets basal metabolic demands but seldom allows surplus accumulation [54].

#### 3.1.2. Influence of the Ecological Context on the Exoskeleton Biochemical Profile

The organic-to-mineral (OM/MM) ratio of the exoskeleton follows the opposite pattern compared to that observed in meat, underscoring a trade-off between structural reinforcement and energetic reserves across *C. sapidus* populations [59]. At Marchica Lagoon, this ratio is markedly higher; that may stem from lagoon conditions that mildly limit calcification or favour a more flexible exoskeleton. Nevertheless, minerals still dominate, ensuring robust protection. This elevated OM/MM ratio signals an extra investment in a compliant, chitin-rich cuticle as well as in protein-dense muscles, an allocation strategy linked to enhanced tolerance of salinity fluctuations and metal stress in portunid crabs [60]. From a food-safety perspective, consuming meat with these features can elevate dietary exposure to tightly bound heavy metals, thereby increasing bioaccumulation risks for mammalian consumers, including humans [61].

The OM/MM ratio in the exoskeleton of individuals from the Moulouya Estuary remained at moderate levels, suggesting a balanced structural response to estuarine dynamic. This reflects the need for both relative flexibility and resistance in response to the sedimentary processes according to the estuarine regimes (e.g., mobility of sedimentary substrata) requiring the resilience and adaptability needed to survive in a large array of different substrata [62,63]. Frequent moulting in a nutrient-rich estuary produces new and wet exoskeletons. Such a pattern is best explained by an accelerated moulting cycle in this highly productive system, where successive new exoskeletons remain thin and hydrated until final hardening, after which available calcium is incorporated to compensate for episodically low salinity [62,63]. In contrast, specimens from Al Hoceima National Park, an area with high salinity and excellent water quality compared to the other investigated areas, exhibited a low exoskeleton OM/MM ratio, pointing to a maximization of biomineralization (calcium carbonate, magnesium) for an optimal exoskeleton calcification to cope with predation and hydrodynamic stress in open coastal waters [64,65]. This is consistent with the ecological requirements associated with a high-energy marine system [64,65]. Here, the crab exoskeleton is dominated by mineral matter as indicated by the OM/MM ratio: it contains more than twice as much calcium carbonate as organic matter, making it the most heavily calcified among all investigated populations. The elevated salinity (~37 PSU) and a plentiful carbonate availability favour complete post-moult calcification, resulting in a thick and rigid exoskeleton that enhances resistance to hydrodynamic stress and predation, albeit at the possible expense of flexibility and overall growth rate [65].

For clarity, the environmental descriptors used in our site-level interpretation are summarised in Materials and Methods (Table S4), linking salinity regime, trophic status, and substrate to the observed contrasts in meat biochemistry and exoskeleton architecture. These findings emphasize the importance of habitat-specific management strategies, particularly in mitigating anthropogenic stressors in transitional zones like estuaries and lagoons. Further research should explore the molecular pathways that mediate these biochemical trade-offs and their implications for coastal ecosystem resilience [41].

### 3.2. Chemical Extraction and Characterization of Chitin and Chitosan

#### 3.2.1. Chitin

Variations in chitin-extraction yield across the three collection sites appear to stem from both intrinsic factors—such as exoskeleton architectures, particularly the organic-to-mineral ratio and the thickness of the chitin–protein matrix—and extrinsic environmental conditions that influence biomineralization. In particular, specimens from Marchica Lagoon produce the highest amount of chitin, whereas those from Al Hoceima National Park give the lowest. Even so, all three yields fall comfortably within the typical range reported for marine brachyuran crabs [66,67]. Crustacean exoskeletons generally contain 10–15% chitin on dry weight, depending on species and environmental conditions [68,69], with the remainder composed mainly of minerals and structural proteins. This compositional pattern is consistent with the exoskeleton of the blue crab [66,67]. Therefore, *C. sapidus* collected from these sites represents a particularly promising raw material for chitin extraction. A higher chitin yield from a given exoskeleton usually indicates a greater organic-to-mineral ratio—that is, a thicker chitin–protein matrix relative to the calcium carbonate phase. The mineral-to-organic ratio is the principal determinant of cost-effective valorisation of *C. sapidus* exoskeletons: a high organic fraction streamlines chitin extraction because less calcium carbonate needs to be dissolved during acid demineralisation [70,71]. However, the high carbonate purity of these exoskeletons renders them an attractive natural source of biogenic CaCO_3_ for animal-feed formulations and nutraceutical tablets [72].

From a processing standpoint and as the variation we deduced for meat from the three sites, the organic-to-mineral (OM/MM) ratio controls reagent demand and effluent load during demineralization/deproteinization: a higher organic content (higher chitin fraction) means less CaCO_3_ to dissolve, reducing acid/base use, time, and saline brines, whereas mineral-rich shells increase operational costs and wastewater volumes [68,70,71]. In our material, lagoon/estuarine *C. sapidus* (Marchica > Moulouya) are the most cost-effective feedstock for chitin–chitosan manufacture, while marine exoskeletons (Al Hoceima)—though still suitable for chitin—are particularly attractive as a biogenic CaCO_3_ co-product for feed premixes and nutraceutical tablets [72]. The site-derived chitosans show high DDA (~79–84%), supporting biomedical applications (wound dressings, drug delivery), antimicrobial packaging films, and water treatment sorbents/flocculants [73,74,75,76]. Compared with pink shrimp (*Parapenaeus longirostris*) exoskeletons used locally (≈24% chitin, dry basis) [77,78,79], the invasive *C. sapidus* is abundant and low value at landing, offering lower raw material costs. Relative to the other invasive crab *Portunus segnis*, both yield high-quality α-chitin/chitosan with similar DDA ranges suited to food-packaging antimicrobials and water treatment adsorbents; *C. sapidus* additionally shows strong antioxidant/biomedical potential [80,81]. Overall, lagoon/estuarine *C. sapidus* is preferred for efficient chitin–chitosan production and downstream biomedical/packaging/remediation markets, whereas marine *C. sapidus* is strategic when a CaCO_3_ co-product is prioritized.

Environmental conditions play a significant role in modulating this balance. Experimental studies on *C. sapidus* have shown that reduced salinity or a lower concentration of dissolved calcium in the water can slow post-moult calcification [63]. As a result, less calcium carbonate (CaCO_3_) is deposited in the cuticle, and the relative proportion of organic matter increases. Crabs from Marchica may be exposed to such conditions, which either inhibit calcification or support an augmented amount of the organic matrix, leading to exoskeletons with a relatively higher chitin-to-calcite ratio. Conversely, the Moulouya and Al Hoceima ones may provide mineral-rich or calcification-favourable conditions, resulting in exoskeletons with an increased inorganic fraction and, therefore, a lower chitin fraction. In this context, the X-ray diffraction crystal characterization (XRD) represents a powerful tool to identify the chitin form and assess its crystallinity and crystallite size. The diffraction profiles of all samples display two dominant reflections at 2θ ≈ 9.6° and 19.6°, accompanied by several weaker features at higher angles, a modest shoulder near 12.7°, and minor peaks centred at roughly 23° and 26.6°. Crustacean-derived α-chitin, in particular, is characterised by intense peaks at ~9° and ~19° 2θ, attributable to the (020) and (110) crystallographic planes, respectively, together with subsidiary reflections in the 12–13°, 21–23°, and 26° regions [82]. This reflection pattern is diagnostic of α-chitin [83]. In the X-ray diffraction (XRD), the pattern of chitin extracted from *C. sapidus* has its dominant peak at 2θ = 19.6°, which is consistent with the literature values [84]. Based on the XRD patterns, a quantitative analysis of crystallinity shows significant differences between all the studied samples. The findings showed that the chitin degree of crystallinity was the highest in Al Hoceima, while chitin extracted by individuals from Marchica and Mouloya have more amorphous content and lower crystallinity. Because all samples were processed under an identical extraction protocol, we attribute the site-dependent differences in crystallinity primarily to environmental/biological factors (e.g., salinity, Ca^2+^ availability, trace-metal burden) while acknowledging that extraction can impact chitin’s molecular structure and influence crystallinity in principle [85].

The three sites’ α-chitin FTIR spectra were similar, with the main differences occurring in the wave number bands, and confirmed that the extracted chitin was protein-free, as evidenced by the peak at 1450 cm^−1^, consistent with findings from Alimi et al. [34]. Wave number variations in FTIR spectra of *C. sapidus* α-chitin and commercial α-chitin were ascribed to ecological and environmental factors that affected the crystalline structure and degree of acetylation [86].

All four patterns of mineral-phase signatures and demineralisation efficiency revealed by XRD show sharp maxima at 2θ ≈ 39.2°, 43–44°, 64°, and 77°; in the untreated control (T), the lines at ≈39° and ≈44° are especially intense. These positions coincide with the (113) and (202) Bragg planes of calcite, while the weaker lines at ≈ 64° and ≈ 77° match the (214) and (300) planes, respectively [87,88]. Standard reference patterns for calcite (JCPDS 05-0586) list the full peak suite 23.1°, 29.4°, 36.0°, 39.4°, 43.2°, 47.5°, 48.5°, 56.6°, 64.0°, and 75.1° 2θ when Cu Kα radiation is used, matching the ensemble observed here [87,89]. The blue crab cuticle is an organic–inorganic composite comprising 15–40% α-chitin/protein embedded in 40–60% CaCO_3_, predominantly as calcite with subordinate aragonite [64]. The control sample T, analysed as an essentially raw exoskeleton, retains the full calcite fingerprint; note the prominent 39° and 43–44° peaks and a detectable shoulder at 29.4° (whereas samples of Marchica Lagoon and Al Hoceima National Park exhibit only faint vestiges of these reflections), indicating that acid treatment removed most of the carbonate phase. Such selective attenuation of mineral peaks after HCl or citric-acid demineralisation is routinely reported for crustacean chitin extractions [90]. Because calcite reflections overlap the high-angle region of the chitin pattern, quantitative parameters such as crystallinity index or Scherrer domain size should be extracted exclusively from the low-angle α-chitin peaks (≈9° and 19° 2θ). Residual carbonate, if ignored, can inflate baseline intensities and bias crystallinity downward [64]. Accordingly, the near absence of carbonate lines in Marchica and Al Hoceima confirms that the demineralisation protocol was sufficient for reliable chitin crystallographic characterisation, whereas any structural metrics derived for the control sample must be interpreted as those of a composite chitin–calcite system.

The crystallinity indices (CrIs) of the blue crab chitin samples range from roughly 60% to almost 90%. Such values lie squarely within the wide span reported for biogenic α-chitin (≈40–90%) and overlap the more common 60–80% band noted for most crustacean and insect sources. By way of comparison, crab exoskeleton α-chitin demineralised with moderate acid treatment typically exhibits a Segal CrI of 75–85% [91]. Collectively, these results show that the extraction protocol preserved, or even enhanced, the crystalline order in individuals from Marchica Lagoon and Al Hoceima National Park, while those of Moulouya Estuary retained a higher amorphous content, underscoring the influence of site-specific biology and processing history on the ultimate structural order of α-chitin.

The X-ray parameters reveal marked differences in long-range order and lamellar length that can be traced to site history and extraction efficiency. The divergence between individuals of Marchica Lagoon and Moulouya Estuary on the one hand and those of Al Hoceima National Park on the other hand implies preferential lateral ordering within sheets characterized by an anisotropic growth gradient likely driven by site-specific biochemical environments during exoskeleton formation [65,92]. For Al Hoceima National Park samples, the largest crystallite sizes were obtained for the reflection showing the strongest site contrast, better long-range order. This pattern is consistent with modest differences in mineralization rate or microstrain during cuticle formation. For the control T, the lower order may be due to species-dependent chitin–protein interactions or to the more severe pretreatment protocol to which the pink shrimp exoskeletons were exposed.

Environmental and biological conditions at the collection sites provide a plausible explanation for the divergent structural signatures observed among the three acid-demineralised blue crab chitin samples. In particular, Moulouya Estuary [52] and Marchica Lagoon [93,94,95] are repeatedly documented as hotspots for heavy metal enrichment and high mineralisation of the water column, factors known to disturb crustacean biomineralization processes and to embed contaminants within the exoskeletal matrix. Elevated trace-metal loads (e.g., Cu and Cd) can be co-precipitated with CaCO_3_ during exoskeleton formation, generating lattice strain and creating amorphous pockets within the chitin–protein network [96]. Such micro-strain broadens X-ray peaks and reduces both the crystallinity index and Scherrer domain length, coherently accounting for the lower CrI (~ 84%) and the smallest coherent lamellae recorded for Marchica Lagoon and Moulouya Estuary. Al Hoceima National Park generally falls between chitins of crabs from Marchica Lagoon and Moulouya Estuary but surpasses both on (110) (6.3 nm). Enhanced coherence along this diagonal may result from subtle variations in mineralisation kinetics or environmental pressures at the collection site. This suggests that exoskeletons developed larger uninterrupted lamellae but still host residual micro-strain, perhaps from a slightly higher organic-to-mineral ratio that leaves some disordered interfaces after acid removal.

The degree of acetylation (DA) showed a distinct inter-site variation, which is the most important parameter in characterizing chitin quality [34]. Together with the good crystallinity and purity, the *C. sapidus* chitin produced in this study can act as a sustainable source for a wide range of high-valued applications in industrial and pharmaceutical uses [33]. These physiochemical characteristics render it an ideal candidate for use related to food end products, biomedical applications, and wastewater treatment adsorbents [97,98].

#### 3.2.2. Chitosan

After alkaline deacetylation of the site-specific chitin, conversion efficiencies remained uniformly high (~79–84%). The greatest yield was obtained from crabs collected in the Moulouya Estuary (84.12% of the initial chitin converted to chitosan), followed by Marchica Lagoon (80.45%) and Al Hoceima National Park (78.75%). These values fall squarely within the 75–85% range typically reported for marine brachyurans and also for *C. sapidus* (i.e., chitosan yields 76–82%) [99]. The marginal differences among sites—slightly higher conversion at Moulouya and slightly lower at Al Hoceima—likely reflect minor variations in reaction conditions or subtle differences in chitin quality rather than fundamental divergences in deacetylation chemistry. Overall, the results indicate consistently efficient chitosan production from all three Moroccan blue crab populations.

The degree of deacetylation (DDA) of chitosan is an important parameter, which affects its solubility and function. These DDAs exceed the 70–75% threshold required for good acid solubility and strong cationic functionality [73], indicating that the alkaline treatment almost completely removed acetyl groups from the crab exoskeleton chitin. By contrast, the control sample (T) retains a DDA of only 61.2%. This places T at the lower bound of what is typically considered chitosan and is consistent with a single alkaline cycle applied to a highly crystalline α-chitin substrate. Accordingly, “T” is expected to be only sparingly soluble in weak acids, to carry a lower density of protonatable amines, and therefore to exhibit reduced intrinsic antimicrobial activity and comparatively higher viscosity relative to the high-DD field samples. We included T as a process/analytical control to benchmark our deacetylation protocol and to validate the FTIR-based DDA determination (Table 5). The data confirm that the site-derived chitosan were well within the performance window for biomedical, antimicrobial, or chelation applications, while the commercial reference would need further deacetylation to reach comparable functionality. Generally, any chitin deacetylated beyond ~50% is termed chitosan [100], for different applications [101], becoming soluble in dilute acids by the addition of numerous free amino groups for chemical interactions and modifications. These results indicate that the chitosan obtained from blue crab exoskeletons collected at Marchica Lagoon and Al Hoceima National Park dissolves readily in mild acidic solutions, whereas the control (T)—chitin of *Parapenaeus*, whose degree of deacetylation is markedly lower—is expected to be only sparsely soluble, displaying behaviour closer to that of the native chitin [74]. High DDA also correlates with enhanced bioactivity: the literature indicates that increasing deacetylation strengthens chitosan’s antimicrobial and other biofunctional activities [73,75]. Thus, crab samples from Marchica (DDA 79.4%) have the most potent bioactivity and surpass the 75% DDA percent threshold that is often quoted for increased bioactivity and the medical grade for chitosan [75], which is suitable for applications which demand maximized functional amine content (e.g., biosensors for enzyme immobilization or advanced wound dressing). In contrast, the DDA of crab samples from Moulouya and Al Hoceima (DDA ~80%) are close to some common commercial food-grade chitosan [76] and should provide sufficient solubility and reactivity for uses in environmental remediation (e.g., heavy metal sorbents) or the food/agro-industrial sector (e.g., antimicrobial packaging films or plant-coating agents).

When comparing data from *C. sapidus* to *Portunus segnis*, the other invasive crab in the Mediterranean, the degree of deacetylation (DDA) of *C. sapidus* chitosan is typically high (~65–82%) [80], with a similar value range reported for *P. segnis* (~75–81%) [81]. Both species yield α-chitin of high crystallinity [102,103], and their deacetylated chitosan retains nanocrystalline domains (~5–6 nm) [103]. Moreover, a less deacetylated *P. segnis* chitosan (DDA ~53%) shows a markedly lower solubility (~53% in dilute acid) and darker colour compared to a purer commercial sample [104], whereas thoroughly deacetylated *C. sapidus* chitosan is more soluble and amenable to further purification (yielding a nearly white product) [105].

The functional properties of both are excellent: *C. sapidus* chitosan (with DDA ~81%) exhibited strong antioxidant activity and falls within the recommended DDA range for biomedical use (80–95%) [80], making it highly suitable for biomedical applications (e.g., wound dressings, drug delivery). *Portunus segnis* chitosan exhibits antimicrobial activities for a broad range of pathogens [66,71] to back uses in food preservation (an antimicrobial coating or film) and water treatment (as a flocculant/ adsorbent in the clean-up of wastewater) [104].

## 4. Materials and Methods

### 4.1. Sampling Localities and Species Identification

**Figure 5:**
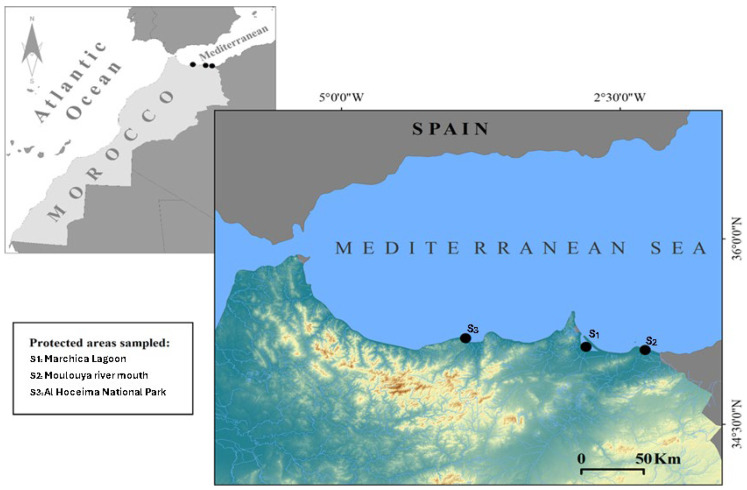
American blue crab sampling sites in northeastern Morocco.

Blue crab samples were collected during 2023–2024 in intertidal to shallow-subtidal habitats (0.5–1 m) using hand nets and fishing pots in three SBEI areas (Figure 5) characterized by different environmental features, namely: (i) the Marchica Lagoon of Nador (S_1_: 35.1550 N, −2.8628 W), a semi-enclosed coastal lagoon with fluctuating salinity (~22–39 PSU), meso- to eutrophic conditions with frequent resuspension of organic detritus, fine sediment substrates, and documented hotspots of trace-metal contamination in sediments/biota [50,51,93,94,95]; (ii) the river mouth swamp of Oued Moulouya (S_2_: 35.1235 N, −2.3415 W), a dynamic river–sea mixing zone (~30–35 PSU brackish–marine) with nutrient pulses and organic-rich silts delivered by the watershed; trace-metal inputs of riverine/agricultural origin are reported for surface sediments [52,53]; and (iii) the marine bay of Al Hoceima National Park (S_3_: 35.1497 N, −4.3668 W), an open, high-energy Mediterranean setting with stable marine salinity (~37 PSU), rocky/reef and seagrass habitats, comparatively low anthropogenic pressure, and overall good ecological status [56,57]. Soon after collection, crab species were placed into single plastic bags kept in cold storage containers filled with ice and then transferred to the laboratory for species identification based on their morphological features.

Blue crabs were identified as *Callinectes sapidus* Rathbun, 1896, mostly paying attention to: (i) the absence of the inner spine in the carpus of the cheliped, a character that allows an easy distinction with respect to *Portunus segnis* (Forskål, 1775), the other blue crab invading the Mediterranean Sea [106]; and (ii) the presence of two strong triangular teeth only, with rounded or acuminate apices, a characteristic that allows an easy identification of *C. sapidus* when compared with the worldwide congeneric species [107]. For each crab we recorded their sex using abdominal morphology (narrow, T-shaped apron in males; broad, U-shaped apron in females) and chelae coloration (bright blue in males; red/orange in females), following standard keys [29,66]. At capture, carapace condition was assessed for hardness by manual palpation: soft-shell/post-moult crabs were excluded, and only hard-shell (intermoult) adults were retained for analyses [17]. We also excluded females, as well as specimens with major damages or limb autotomy.

### 4.2. Preparation and Processing of the Crab Tissues

Blue crab individuals were first scraped and thoroughly washed with distilled water to remove external epiphytes, then weighed (total body weight) with a OHAUS Scout (SP401, Parsippany, NJ, USA) Pro balance (accuracy 0.1 g), measured for their exoskeleton width (including spines) with a sliding calliper (accuracy 1 mm), and finally frozen at −20 °C. Among the sampled material, 33 large individuals (ranging from ~10 to 17 cm in exoskeleton width and ~110 to 300 g in total body weight) of *C. sapidus* were selected from each collection site. The specimens were thawed sequentially, in small batches (≤13 individuals) and by sites, and left to dry at room temperature, under controlled conditions. Then, the meat and exoskeleton were mechanically separated with laboratory scissors, pliers, scalpels, and spatulas. The raw meat was cleaned with distilled water, blotted dry, and stored at 4 °C until it was used for the subsequent laboratory steps—within 48 hours (h). The exoskeletons were cleaned with distilled water to remove any eventual residual tissue and dried at room temperature to eliminate surface moisture. Finally, both the raw meat and exoskeleton originating from the same sampling areas were chopped, mixed to ensure consistency, and weighted. The mixing of samples from the same area is chosen to investigate the population variations in tissue composition. A total of 100 g of samples from the different pools obtained was then isolated to be used for the subsequent analyses (carried out in 3 replicates each).

Ninety-nine blue crab specimens were collected from three Moroccan sites—Marchica Lagoon (S_1_), the Moulouya River Estuary (S_2_), and Al Hoceima National Park (S_3_).

### 4.3. Physical Characterization of the Samples

#### 4.3.1. Dry Matter Determination

To determine the dry matter content, the AOAC Official Method 930.15 for loss on drying (moisture) in feeds was applied [108]. Separate analyses were performed on raw meat and air-dried exoskeletons. A total of 3 g was dried at 105 °C for 2 h in a Memmert UN110 (Memmert GmbH, Schwabach, Germany) forced draft oven and subsequently moved to a Brand 65231 (Germany) desiccator to cool to room temperature after drying. A Sartorius ED822-CW ((Sartorius AG, Göttingen, Germany) analytical balance was finally used to measure the sample’s dry mass (M_d_). The percentage of dry matter was calculated following the subsequent formula [109]:

(M_d_)/M_f_ × 100

where: M_f_: fresh mass (g); M_d_: dry mass (g).

#### 4.3.2. Organic and Inorganic Matter Determination

To determine the organic and inorganic matter content, the method described by Gökoðlu and Yerlikaya [110] was used. Separate analyses were performed on the oven-dried meat and oven-dried exoskeletons described in the Section 2.3.1.

Specifically, the dry sample was subjected to calcination at 550 °C for 5 h in a muffle furnace (Protherm PLF 120/7, Ankara, Turk). Following the complete combustion of organic components, the residual ash was weighted to obtain values of inorganic matter, whereas the weight loss of the sample was used to calculate the organic fraction and thus the amount of organic matter. In general, the percentage of organic matter was calculated following the subsequent formula:

(Dry Weight − Ash Weight/Dry Weight) × 100

### 4.4. Biochemical Characterization of Samples

#### 4.4.1. Preparation of Extracts (Reducing Sugars, Proteins, and Lipids)

To obtain sugar extracts, 5 g of raw meat was homogenized in 20 millilitres (mL) of a 70% ethanol solution in distilled water. To obtain protein extracts, 5 g of raw meat was homogenized in 50 mL of a 0.15 M sodium chloride solution in distilled water. Both mixtures were subsequently immerged in an ice bath for 10 min and then, respectively, centrifuged at 10,000 and 12,000 rpm at 4 °C for 20 min. In both cases, the supernatant was finally isolated for the subsequent steps [111,112]. The whole procedures were collected for downstream assays. Per individual, downstream volumes were as follows: reducing sugars (Fehling titration), 1.0 mL per replicate (technical triplicates; total ethanol supernatant reserved ≈ 5 mL including blanks/standards); proteins (Kjeldahl), 2.0 mL (≈2.0 g) per replicate (technical triplicates; total saline supernatant reserved ≈ 6 mL). Extract preparation was scaled accordingly; any remaining supernatant was stored at 4 °C and used within 24–48 h.

To obtain lipid extracts, 5 g of raw meat was oven-dried at 50 °C for 8 h to ensure complete dehydration. The dried meat was finely ground with the electric grinder and then subjected to the Soxhlet extraction using 150 mL of chloroform/methanol 2:1 (*v*/*v*) ratio at 55–60 °C for 24 h. At the end of the extraction, the solvent containing the dissolved lipids was removed using a rotary evaporator (Model RE-2000A, Lab-Kits, Beijing, China) under reduced pressure at a temperature of 40–45 °C. The dried lipid residue was stored in amber vials under nitrogen gas at 4 °C to prevent oxidation until the subsequent steps [113,114].

#### 4.4.2. Reducing Sugars

To quantify the reducing sugars content, the Fehling’s method was used, following the original protocol established by Fehling [115] and standardized procedures outlined in reference analytical chemistry handbooks [116]. Fehling’s solution was prepared by mixing equal volumes of Fehling’s A (dissolving 34.64 g of crystals of copper (II) sulphate pentahydrate in 500 mL of distilled water) and Fehling’s B (dissolving 173 g of potassium sodium tartrate tetrahydrate and 50 g sodium hydroxide in 500 mL of distilled water). A sugar solution was prepared by adding and dissolving 5 g of supernatant in 100 mL of distilled water. Then, 4 mL of Fehling’s solution was added to the sugar solution. The mixture was heated in a boiling water bath (Grant SBB28L Boiling Water Bath, 28L, Grant Instruments, Cambridge, UK) at 100 °C for 10 min until the formation of a reddish-orange precipitate, indicating the presence of reducing sugars. The obtained precipitate was first dried at 50 °C for 12 h in the convection oven and then analysed using Fourier Transform Infrared (FTIR) spectroscopy through a Jasco FT/IR-4700 spectrophotometer (Sunway Scientific Corporation, Taipei, Taiwan). The absorbance was compared to a standard spectrum to identify the distinctive peaks based on their functional groups [117].

#### 4.4.3. Total Nitrogen and Proteins

To quantify the total nitrogen content, the Kjeldahl method was used, employing a Kjeldahl digestion unit (Behrotest Complete System Basic Block Digestion, Behr Labor-Technik GmbH, Düsseldorf, Germany) [118,119]. Two g of supernatant was first mixed with 30 mL of 98% concentrated sulfuric acid and 2 g of a catalyst mixture of potassium sulfate (K_2_SO_4_) and copper (II) sulfate (CuSO_4_) (1:1 (*w*/*w*) ratio), in appropriate tubes. The tubes were then placed into the system’s heating block until a temperature of 360–380 °C was reached and a clear solution was formed, indicating the complete decomposition of organic material. Soon after cooling, the digested content was transferred into a VAPODEST 550 (Königswinter, Germany) steam distillation device to convert the ammonium ions (NH_4_^+^) into ammonia gas (NH_3_) by adding sodium hydroxide (NaOH). A hydrochloric acid solution (HCl) was also progressively introduced. The ammonia gas was distilled into a 2% boric acid solution that was further supplemented with a colour indicator (bromothymol blue). The colour switch of the solution from green to red/orange marked the endpoint of the titration. The VAPODEST 550 monitored the colour change and recorded the final volume of HCl consumed immediately after the NH_3_ had been fully captured into the boric acid solution, ensuring precise neutralization of the ammonia–boric acid complex. Based on that, the total nitrogen concentration was calculated automatically following the subsequent formula [111]:

Total Nitrogen (%) = [(V_HCl_ × N_HCl_) − (V_Blank_ × N_HCl_)/Weight of Sample (g)] × 1.4

where: V_HCl_: volume of hydrochloric acid (HCl) used for titration (mL); N_HCl_: normality of the HCl solution; V_Blank_: volume of HCl used for the blank titration (mL).

Finally, the obtained value of total nitrogen content was converted to protein content (in percentage) using the standard conversion factor of 6.25 [120].

#### 4.4.4. Lipids

To quantify the lipid content, the sulfo-phospho-vanillin reaction was used, following the AOAC Final Action method [121]. A portion (⅖) of the lipid residue previously obtained was first dissolved in 2 mL of chloroform. Then, 2 mL of concentrated sulfuric acid (H_2_SO_4_) was added to 200 µL of the mixture and heated to 100 °C for 10 min. After cooling at room temperature, 5 mL of the sulfo-phospho-vanillin reagent was added, waiting for about 15 min until a colorimetric shift was observed, indicating the presence of lipids. The absorbance of the reaction mixture was measured at 530 nm using a UV-Vis spectrophotometer (Autopol V Polarimeter by Rudolph Research Analytical, Gaithersburg, MD, USA). Lipid concentration was determined by comparing the absorbance of each sample to a standard curve prepared with known lipid concentrations (0.1 to 1 mg/mL) [122].

### 4.5. Statistical Analyses

To compare the values obtained from the physical and the biochemical characterizations of the samples, the R software (version 4.1.3) and the agricolae package were used [123]. We analysed meat yield (%) and exoskeleton yield (%) with site (Marchica Lagoon, Moulouya Estuary, Al Hoceima Bay) as a fixed factor. Replication per site was *n* = 33 for each location (total *n* = 99). Only adults (carapace width ≥ 10 cm; body mass ≥ 140 g) were retained. When the conditions of normality and/or homoscedasticity were fulfilled, ANOVA was performed, followed by Tukey tests. Conversely, when these conditions were not satisfied, a Kruskal–Wallis test was used, followed by Wilcoxon tests. The statistical significance level was set at the 0.05 probability level. Considering the distributional features of the data, these statistical techniques guaranteed a solid assessment of variations across sites [122].

### 4.6. Chitin and Chitosan Extraction and Characterization

#### 4.6.1. Exoskeleton Preparation and Processing

The remaining chopped exoskeletons described in Section 2.2 were first dried at 40 °C for 24 h in a Memmert INE500 (Memmert GmbH, Schwabach, Germany) oven to reduce the moisture content and prevent microbial deterioration. In parallel, a commercial α-chitin (T) standard extracted from *Parapenaeus* shrimps (Darmstadt, Germany, product C30-20 100 MG, CAS 1398-61-4; humidity < 10%; ≥95% purity) was subjected to the same drying regime to ensure identical moisture histories. Both materials were subsequently ground with a Royal Catering RCMZ-800N (Berlin, Germany) electric grinder to produce a homogeneous powder, which was then sieved through a 250 µm mesh to obtain uniform particle sizes.

#### 4.6.2. Chitin Extraction

To obtain the chitin, hydrochloric acid HCl (1N) at a solid-to-liquid ratio of 1:20 (*w*/*v*) was initially added to 30 g of powdered exoskeletons. The suspension was mixed at room temperature for 5 h with a Faithful SH-2 (Ningbo, China) magnetic stirrer and subsequently filtered using a vacuum filtration system (Whatman filter paper) to remove mineral components. To guarantee that the calcium carbonate (CaCO_3_) in the exoskeletons was completely dissolved, the whole demineralization procedure was carried out three times. The resulting material was finally dried in the Memmert INE500 oven at 40 °C for 24 h. The acid-treated material was then weighted, and 15 g was added to a 200 mL solution of sodium hydroxide (NaOH) at a concentration of 3% with a solid-to-liquid ratio of 1:13 (*w*/*v*). The mixture was stirred at 60 °C for 12 h using a heating plate, then filtered once more, and any leftover proteins and excess NaOH were eliminated using distilled water. Subsequently, the samples were treated with a 2% hydrogen peroxide solution (H_2_O_2_), with a solid-to-liquid ratio of 1:50 (*w*/*v*), for 10 min, using the magnetic stirrer to ensure thorough mixing. The solution was then dried for 24 h at 50 °C in the Memmert INE500 oven until its weight remained constant.

#### 4.6.3. Deacetylation of Chitosan

To deacetylate the chitin and obtain the chitosan, 5 g of the chitin described in the Section 4.6.2 was heated in a GEA YX-24HDD autoclave in a 40% NaOH solution at 120 °C for 6 h (volume ratio of 1:20 *w*/*v*). After the mixture had cooled to room temperature, a fine filter was used to separate the solid chitosan. To eliminate residual NaOH and byproducts, the chitosan was washed multiple times with distilled water until the solution dropped to a neutral pH (pH 7.0 ± 0.1) [124,125]. The washed chitosan was then dried for 24 h at 50 °C in the Memmert INE500 oven until its weight remained constant.

#### 4.6.4. Physicochemical Characterization of Chitin and Chitosan

- X-ray diffraction (XRD)

The dried chitin and chitosan described in Section 4.6.2 and Section 4.6.3 were ground manually into a fine powder using an agate mortar and pestle and sieved < 250 µm. To determine the chitin’s crystallinity index (CrI), type, and crystallite size (D), aliquots of 200 mg were analysed on a Shimadzu XRD-6000 diffractometer (Shimadzu Corp., Kyoto, Japan) equipped with Cu Kα radiation (λ = 1.5406 Å). Operating parameters were 40 kV, 40 mA, fixed divergence/slit geometry, and a graphite monochromator. Diffraction patterns were recorded at room temperature from 2θ = 5° to 50° in continuous-scan mode (1° min^−1^, step size = 0.02°). Instrumental broadening was evaluated with a standard LaB_6_ (NIST 660b, NIST, Gaithersburg, MD, USA) reference and subtracted from experimental peak widths prior to crystal-size calculations [126].

- Crystallinity index (CrI)

To determine the crystallinity index, the Segal method described by Neves et al. [126] was employed, using the intensity of the (002) reflection at 2θ ≈ 19.6° and the minimum intensity (I_amorphic_) between the (002) and (110) reflections. CrI was then calculated with the following equation:

CrI = [(I_19.6°_ − I_amorphic_)/I_19.6°_] × 100

where: I_19.6°_ stands for the crystalline peak’s intensity at 2θ and I_amorphic_ represents the intensity of the amorphous region, measured as the lowest intensity between two main crystalline peaks in the XRD diffractogram, indicating the disordered parts of chitin.

The CrI value was then also used to infer the chitin type (α or β), as high values indicate a more crystalline and ordered structure (e.g., chitin α), while low values suggest a more random amorphous arrangement (e.g., chitin β) [105].

- Crystallite size (D)

The average crystallite size was calculated with the Scherrer equation:

D = Kλ/β cosθ

where K = 0.94 (shape factor for plate-like crystals), λ = 1.5406 Å, β is the full width at half maximum (FWHM, in radians) of the (002) peak corrected for instrumental broadening, and θ is the Bragg angle (half of 2θ ≈ 19.6°). Resulting D values are reported as means ± SD of three replicate scans.

- Degree of acetylation (DA) and of deacetylation (DDA)

To determine the structural and functional properties of chitin and chitosan, we investigated the degree of acetylation (DA) for the chitin and of deacetylation (DDA) for the chitosan [127]. A quantity of 2 mg of both powdered samples was time by time mixed with 200 mg of spectroscopic-grade potassium bromide (KBr) to form translucent pellets. For the FTIR analyses, the JASCO FT/IR-4700 spectrophotometer (JASCO Corp., Tokyo, Japan) was operated in the mid-infrared (7800–350 cm^−1^), near-infrared (15,000–2200 cm^−1^), or far-infrared (5000–220 cm^−1^) ranges, depending on the requirements. The DA was calculated using the following formula [34]:

DA (%) = A1620/A3444 × 115 (1)

where: A3444 is the peak absorbance value of the hydroxyl (-COCH_3_) group; and A_1620_ is the peak absorbance value of the amide (-NH_2_) group. The correction factor obtained from standard references is 115 and is used to normalize absorbance values.

The obtained value was then used to calculate the DDA using the following formula [34,128]:

DDA (%) = 100% − 75.19 × DA (%) (2)

where: 75.19 (=1/1.33) is a correction factor obtained from standard references and used to normalize absorbance values.

### 4.7. Environmental Context of Sampling Sites

To support the interpretation of site-specific differences, we compiled a concise environmental profile for each sampling location from peer-reviewed sources cited in this manuscript. For each site, we summarised hydrography, salinity (PSU), trophic status/food availability, substrate/habitat, and trace-metal pressure. When multiple sources were available, we reported ranges or consensus qualitative descriptions, prioritising studies overlapping our sampling period. The descriptors and sources are listed in Table S4 and are cited in the Discussion to relate habitat conditions to meat biochemistry and exoskeleton architecture ([50,51,52,56,57,93,94,95], and to document mechanistic pathways [62,65]).

## 5. Conclusions

The invasive blue crab *Callinectes sapidus* has become both an ecological challenge and a promising raw material for Morocco’s blue economy. Knowledge of the ecological characteristics of the invaded sites and the effects on crab populations represents a crucial step to transform the invasive pest to societal benefits. In the present study, the three investigated Moroccan populations of *C. sapidus* display distinct adaptive strategies that mirror their local environments. Marchica Lagoon, a nutrient-rich lagoon, favours an opportunistic strategy in which crabs maximise somatic growth and lipid storage. Moulouya Estuary, a productive but stress-prone estuary, elicits an acclimatory strategy characterised by rapid moulting and glycogen buffering to cope with fluctuating salinity. Al Hoceima National Park, an oligotrophic, open-coast habitat, promotes a conservative strategy that prioritises a heavily mineralised exoskeleton, slower metabolism, and minimal energy reserves. These site-specific responses underscore the biochemical plasticity of *C. sapidus*, whose tissue composition shifts with habitat conditions, seasonality, and ontogenetic stage. Overall, the edible muscle of *C. sapidus* delivers roughly 18–20% high-quality protein enriched in essential amino acids and ω-3 PUFA, values that rival or surpass many commercial crustaceans.

From a sustainable exploitation point of view, this invasive biomass therefore offers Morocco a new efficient and economically attractive feedstock to exploit. Beyond the use of its meat as a major source of food, the “crab waste” can generate benefits for human society. Indeed, the exoskeleton contains about 55–60% calcium carbonate and 20–25% chitin, making it an efficient and economically attractive feedstock for chitin production without inflating raw materials [23]. Moroccan processors routinely valorise the exoskeletons of the pink shrimp *Parapenaeus* spp., which sell for EUR 0.5–7 kg^−1^ and yield about 24% dry chitin after standard demineralisation and deproteinisation procedures [77]. Because blue crab landings are abundant and readily collected, each 100 g individual can supply roughly sixteen grams of chitin, in line with the extraction yields of the pink shrimp [91,92]. In addition, when compared with the co-familiar Mediterranean-invasive blue crab *Portunus segnis* (Forskål, 1775), *C. sapidus* has higher yields of chitin than *P. segnis* (31.93% vs. 27.53%) and α-chitin in *C. sapidus* is more versatile than its β-form in *P. segnis*, supporting potential wider applicability in biotechnological fields [129,130].

In addition, the chemical deacetylation of this chitin routinely produces chitosan with degrees of deacetylation close to 80%, as shown for Moulouya Estuary samples, meeting specifications for biomedical, active-packaging, and water treatment applications [76]. Because each 100 g crab yields approximately 14–16 g of chitin, seven medium-sized crabs can supply the same polymer mass that would require several hundred pink shrimp (*Parapenaeus* spp.) while matching or exceeding the yields obtained from the already-commercialised *Portunus segnis*, an invasive pest fast spreading along the Tunisian coasts since 2014, depressing the local artisanal fishery. Thanks to FAO programmes and national R&D actions, this invasive pest has been transformed from an ecological threat into a source of local prosperity [104,105]. Similar initiatives should also be encouraged for the species here investigated to exploit this invasive source and mitigate its environmental pressure.

In summary, *C. sapidus* also readily bio-accumulates trace elements in its hepatopancreas, gills, muscle, and even exoskeleton [131]. This trait, well documented in Mediterranean and Atlantic populations, means the crab can function not only as a biologically robust indicator of metal pollution gradients in lagoons and estuaries [132], but also (as its mineralised cuticle actively sorbs cations) as a modest, self-replicating bioremediation agent that helps immobilise contaminants in situ. To realise these dual ecological benefits while safeguarding product safety, site-specific assays of crab flesh and exoskeletons must precede any industrial processing.

## Figures and Tables

**Table 1:** Biochemical composition and organic and inorganic matter contained in the meat and exoskeleton of *C. sapidus* in the three SBEIs (reported in percentages). Replicates per site *n* = 33. Pairwise differences in the figures were tested with Tukey’s HSD (α = 0.05), see also Table S2. DM = dry matter, OM = organic matter, WW = wet weight, MM = mineral matter.

	Meat										Exoskeleton			
	Dry Matter	Organic Matter	Proteins		Lipids		Reducing Sugars		Ash	OM/MM	Dry Matter	Organic Matter	Ash	OM/MM
			(%WW)	(%DM)	(%WW)	(%DM)	(%WW)	(%DM)						
**Marchica** **(S_1_)**	15.38 ±0.49	12.75 ±0.45	3.36 ±0.21	21.87 ±1.15	0.30 ±0.01	1.98 ±0.05	0.09 ±0.02	0.56 ±0.15	2.89 ±0.21	4.41 ±0.36	87.82 ±1.35	37.43 ±1.12	48.52 ±2.05	0.77 ±0.048
**Moulouya** **(S_2_)**	15.64 ±2.85	13.71 ±1.70	2.89 ± 0.55	18.46 ±1.07	0.27 ±0.05	1.73 ±0.09	0.14 ±0.03	0.91 ±0.1	1.67 ±0.58	8.21 ±1.03	75.41 ±0.96	26.89 ±0.29	50.39 ±0.21	0.53 ±0.007
**Al Hoceima** **(S3)**	13.49 ±0.70	12.17 ±0.12	2.31 ±0.20	17.11 ±1.17	0.21 ±0.01	1.56 ±0.04	0.06 ±0.01	0.41 ±0.09	1.32 ±0.06	9.22 ±0.43	93.91 ±0.29	30.74 ±0.47	62.17 ±0.15	0.49 ±0.008

**Table 2:** Yields of chitin extraction and chitosan conversion from *C. sapidus* exoskeletons collected at different sites. ^1^ Chitin yield (%) = (mass of extracted chitin/dry exoskeleton mass) × 100. ^2^ Chitosan yield (%) = (mass of chitosan obtained from extracted chitin/mass of extracted chitin) × 100. ^3^ Chitosan yield (%) of total crab mass calculated by multiplying the chitin yield by the chitosan conversion efficiency, based on average crab exoskeleton yield and mass. ^4^ Approximate value for control sample (T) calculated based on average conversion efficiencies observed for S_1_–S_3_.

	Chitin Yield (% of Dry Exoskeleton Mass) ^1^	Chitosan Yield (% of Extracted Chitin) ^2^	Chitosan Yield (% of Total Crab Mass) ^3^
**Marchica**	17.28%	80.45%	15.67%
**Moulouya**	12.23%	84.12%	13.59%
**Al Hoceima**	10.30%	78.75%	14.50%
**T** **(Control)**	N/A	≈80%	N/A
**Mean ± SD**	13.27 ± 2.54%	81.11 ± 2.75% ^4^	14.59 ± 1.04%

**Table 3:** X-ray-based structural parameters of chitin samples related to crystallinity index (CrI) and crystallite size (D). * Type inferred from CrI > 70% (highly ordered antiparallel α-chitin lattice).

Crystallinity Index (CrI)					Crystallite Size (D)				
					(20)	(110)	(120)	(130)	Miller Plane
	2θ [°]	I19.6°	I_amorphic_	CrI % *	9.4	19.3	20.7	23.3	Nominal 2θ (°)
					Lowest	Moderate	Highest	High	Packing Density
**S_1_**	19.38	1130	176	84.42	8.9	5.4	6.6	3.5	**D_value_ (nm)**
**S_2_**	19.66	1000	158	84.20	7.4	5.6	7.1	5.7	
**S_3_**	19.66	882	168	80.95	8.7	6.3	5.7	3.5	
**T**	19.36	556	144	74.10	6.7	4.6	4.0	3.5	

**Table 4:** Degrees of acetylation (DAs) of chitin of *C. sapidus* samples from the three SBEI areas.

	% Transmittance of Amide Groups (1619–1620)	% Transmittance of Hydroxyl Groups -OH (3443–3245)	Absorbance of 1619–1620 Amide Groups	Absorbance of Hydroxyl Groups -OH (3443–3245)	DA
	-N-C=O		-N-C=O		
**S_1_**	48.551	42.995	0.361	0.351	77.36
**S_2_**	63.261	54.520	0.257	0.226	85.52
**S_3_**	61.982	51.384	0.276	0.291	71.21
**T**	86.301	86.605	0.064	0.070	68.41

**Table 5:** Degrees of deacetylation of chitosan of *C. sapidus* samples from the three SBEI areas.

	Absorbance of 1620 Amide Groups	Absorbance of Hydroxyl Groups -OH (3441)	DDA
	-N-C=O		
**S_1_**	0.00811	0.02960	79.4
**S_2_**	0.00979	0.03437	78.5
**S_3_**	0.01524	0.04527	74.6
**T**	0.02909	0.05650	61.2

## Data Availability

Data will be made available on request.

## Supplementary Materials

The following supporting information can be downloaded at: https://www.mdpi.com/article/10.3390/md23090367/s1, Table S1: Sampling of *C. sapidus* in the three protected areas (SBEIs); Table S2: Results of one-way ANOVA testing for differences in meat and exoskeleton biochemical variables of *Callinectes sapidus* among sampling sites; Table S3: Allocation of relevant bands in the FTIR spectra of chitin isolated from pink shrimp and *C. sapidus* α-chitin within the three SBEI areas; Table S4: Environmental context of the three sampling sites.
