# Supplementary material for: Meat Nutritional Value and Exoskeleton Valorisation of Callinectes sapidus from Three Sites of Biological and Ecological Interest in Morocco: Scientific Insights Toward a Management Strategy in the Mediterranean Sea
Source: Mar Drugs. 2025 Sep 21;23(9):367. doi: 10.3390/md23090367 (PMC12709225; doi:10.3390/md23090367)
Supplement: Supplementary file 1 [file marinedrugs-23-00367-s001.zip › marinedrugs-3863726-supplementary.pdf]

Table S1. Sampling of *C. sapidus* in the three protected areas (SBEI)

specimen	*C. sapidus* Lagoon (S_1: 35,1550 N; -2,8628 W)							*C. sapidus* Estuary (S_2: 35,1235 N; -2,3415 W)							*C. sapidus* AHNP(S_3: 35,1497;N; -4,3668W)						
	Sex	Total weight (g)	Raw meat (%)	Raw meat weight (g)	Raw carapace weight (g)	Carapace (%)	Measure (cm)	sex	Total weight (g)	Raw meat (%)	Raw meat weight (g)	Raw carapace weight (g)	Carapace (%)	Measure (cm)	sexe	Total weight (g)	Raw meat (%)	Raw meat weight (g)	Raw carapace weight (g)	Carapace (%)	Measure (cm)
1	F	232.58	23.99	55.80	119.38	51.33	13.50	F	262.76	22.42	58.91	129.88	49.43	13.40	F	242.00	22.54	54.55	108.27	44.74	14.60
2	F	245.89	24.70	60.73	124.91	50.80	13.70	M	240.00	24.76	59.42	132.94	55.39	14.00	F	256.00	22.56	57.75	115.74	45.21	16.40
3	M	287.56	24.20	69.59	114.97	39.98	13.70	M	240.97	24.78	59.71	132.90	55.15	15.40	F	240.00	22.58	54.19	109.49	45.62	16.60
4	M	309.80	25.70	79.62	172.50	55.68	17.00	F	312.81	23.80	74.45	171.70	54.89	17.80	M	190.00	23.60	44.84	87.29	45.94	13.20
5	F	198.80	25.61	50.91	84.79	42.65	12.50	F	251.77	24.82	62.49	134.93	53.59	13.00	F	240.00	23.62	56.69	132.38	55.16	17.30
6	M	205.10	23.45	48.10	110.98	54.11	12.00	F	319.73	22.86	73.09	167.15	52.28	17.40	M	198.00	22.64	44.83	91.61	46.27	13.70
7	M	207.10	25.45	52.71	110.98	53.59	12.60	M	293.70	21.88	64.26	158.51	53.97	14.40	F	110.00	21.66	23.83	50.89	46.26	10.00
8	F	231.20	23.70	54.79	117.96	51.02	13.60	M	314.66	22.92	72.12	168.94	53.69	15.10	M	214.00	22.68	48.54	98.74	46.14	13.30
9	F	303.10	22.99	69.68	162.31	53.55	17.90	F	195.63	24.92	48.75	104.58	53.46	14.40	F	198.00	23.70	46.93	90.94	45.93	12.10
10	M	300.90	24.80	74.62	170.94	56.81	15.50	M	314.21	20.94	65.80	161.13	51.28	13.70	M	200.00	23.72	47.44	95.28	47.64	15.70
11	F	215.90	22.08	47.67	92.34	42.77	12.20	M	316.55	24.94	78.95	168.29	53.16	16.50	M	188.00	25.74	48.39	85.16	45.30	13.80
12	M	216.86	24.10	52.26	91.82	42.34	12.30	F	282.84	25.76	72.86	150.20	53.10	14.60	F	222.00	22.76	50.53	99.79	44.95	13.20
13	M	217.83	24.12	52.54	91.40	41.96	16.50	M	272.65	25.78	70.29	144.77	53.10	14.10	M	190.00	22.78	43.28	84.78	44.62	12.00
14	F	218.79	24.14	52.82	91.10	41.64	12.60	M	304.12	25.80	78.46	152.51	50.15	16.40	M	180.00	21.80	39.24	93.02	51.68	11.60
15	M	219.76	24.16	53.09	90.96	41.39	12.00	M	198.65	16.83	33.43	105.79	53.25	12.40	F	210.00	22.82	47.92	108.86	51.84	12.10
16	M	217.72	24.18	52.64	89.68	41.19	12.30	F	270.06	17.79	48.04	144.24	53.41	14.00	M	280.00	23.84	66.75	145.66	52.02	11.50
17	F	221.68	24.20	53.65	90.93	41.02	12.20	M	260.71	18.76	48.91	139.78	53.62	12.20	M	174.00	19.86	34.56	90.85	52.21	12.30
18	M	222.65	24.22	53.93	111.08	49.89	12.10	F	166.31	19.72	32.80	89.58	53.86	11.60	M	168.00	22.88	38.44	88.12	52.45	11.60
19	M	223.61	24.08	53.85	91.23	40.80	13.30	M	232.67	20.69	48.14	125.98	54.15	14.40	F	182.00	21.90	39.86	95.99	52.74	11.30
20	F	224.58	24.10	54.12	91.58	40.78	12.70	F	310.49	21.65	67.22	169.08	54.46	17.00	M	286.00	24.92	71.27	151.75	53.06	16.50

21	M	215.54	24.12	51.99	108.46	50.32	11.30	M	228.43	22.61	51.65	105.04	45.98	15.30	M	180.00	22.94	41.29	96.17	53.43	12.20
22	M	226.51	24.14	54.68	92.85	40.99	12.20	M	216.12	23.58	50.96	100.00	46.27	14.00	F	162.00	22.96	37.20	87.17	53.81	11.00
23	F	232.00	24.16	56.05	95.75	41.27	13.70	M	218.43	24.54	53.60	101.57	46.50	12.20	M	158.00	22.98	36.31	85.62	54.19	11.50
24	M	230.66	24.18	55.77	96.05	41.64	14.70	M	198.09	25.51	50.53	92.47	46.68	14.40	M	192.00	23.00	44.16	104.72	54.54	12.90
25	F	228.01	23.20	52.90	118.82	52.11	13.20	F	188.53	26.47	49.90	88.23	46.80	11.40	F	196.00	24.02	47.08	107.53	54.86	14.20
26	F	216.00	24.22	52.32	91.26	42.25	17.40	M	176.88	27.44	48.54	82.94	46.89	12.90	F	182.00	23.04	41.93	100.37	55.15	13.70
27	M	218.87	25.82	56.51	94.38	43.12	11.60	M	287.25	24.84	71.35	134.86	46.95	14.80	F	170.00	23.06	39.20	77.18	45.40	12.50
28	F	198.60	25.84	51.32	86.79	43.70	11.20	M	288.22	24.86	71.65	135.51	47.02	12.30	M	160.00	23.08	36.93	89.01	55.63	12.10
29	M	188.90	25.86	48.85	83.55	44.23	11.40	F	289.18	19.88	57.49	141.96	49.09	16.00	M	164.00	24.80	40.67	75.16	45.83	11.60
30	F	176.00	25.88	45.55	78.72	44.73	16.40	M	290.14	24.90	72.24	136.89	47.18	13.00	F	162.00	24.80	40.18	72.92	45.01	11.30
31	M	243.86	25.90	63.16	133.15	54.60	13.80	M	291.11	23.92	69.63	146.43	50.30	16.40	M	146.00	22.48	32.82	63.03	43.17	10.00
32	F	244.54	25.92	63.38	131.17	53.64	13.50	F	214.82	20.94	44.98	101.89	47.43	14.00	M	150.65	21.68	32.66	69.27	45.98	10.50
33	M	225.78	23.70	53.51	118.76	52.60	13.44	F	216.99	21.98	47.69	109.38	50.41	14.20	F	149.50	22.65	33.86	67.19	44.94	10.50

Table S2. Results of one-way ANOVA testing for differences in meat and exoskeleton biochemical variables of Callinectes sapidus among sampling sites

	Variable	Units	df_between	df_within	F	p_value
Meat	Dry matter	% WW	2	96	15.4	1.58e-06
	Organic matter	% WW			19.28	9.17e-08
	Proteins	% DM			155.3	1.11e-16
	Lipids	% DM			362.19	1.11e-16
	Reducing sugars	% DM			160.53	1.11e-16
	Ash	% WW			175.09	1.11e-16
	OM/MM	ratio			463.02	1.11e-16
Exoskeleton	Dry matter	% DM	2	96	3111.59	1.11e-16
	Organic matter	% DM			1805.86	1.11e-16
	Ash	% DM			1269.99	1.11e-16
	OM/MM	ratio			939.35	1.11e-16

Table 3. Allocation of relevant bands in the FTIR spectra of reference chitin and *C. sapidus* α-chitin within the three SBEI areas

**Assignments**	**wavenumber (cm^{-1})**			
	**Commercial α-chitin**	**S_1 α-chitin**	**S_2 α-chitin**	**S_3 α-chitin**
ν(OH)	3441	3441	3443	3445
ν(CH_2)	2890	2897	2901	2895
ν(C=O) amide I	1619	1620	1619	1619
ν(CH3) amide III)	1394	1353	1361	1362
ν (Cycle pyranose)	1282	1309	1307	1309
ν(C–O-C) Glycosidic linkage)	1168–1189	1118–1152	1112–1151	1120–1157
Δ of the CH_3. COH group)	548	525	519	522

ν: stretch; *Δ*: bending.

Table 4. Environmental context of the three sampling sites

Parameter	Marchica Lagoon (S1)	Moulouya Estuary (S2)	Al Hoceima Bay / AHNP (S3)	Sources
Hydrography	Semi-enclosed coastal lagoon with restricted inlet and long residence times	River–sea mixing zone at the Moulouya mouth; highly dynamic	Open, high-energy Mediterranean bay within a marine protected area; low anthropogenic pressure	Marchica: [50,51,93–95] Moulouya: [52] Al Hoceima: [56,57]
Salinity (PSU)	22–39, fluctuating with inlet exchange and watershed inputs	~30–35 (brackish–marine), varies with discharge and tides	~37 (stable marine)	
Trophic status / food availability	Mesotrophic–eutrophic; frequent resuspension of organic detritus/benthic prey	Productive estuary; nutrient pulses during floods; abundant detritus	Oligotrophic, clear waters; lower prey density	
Substrate / habitat	Fine sediments; resuspension of silt/clay	Fine, organic-rich silts; shifting beds	Rocky reefs, coarse sands, seagrass meadows	
Trace-metal pressure (Cd, Pb, Cu, Zn)	Elevated; hotspot documented in sediments/biota	Elevated; river-borne/agricultural inputs	Low–background relative to S1–S2	
Carbonate/alkalinity context (relevance to calcification)	Variable; low-salinity episodes can transiently lower Ca^{2+}/alkalinity	Variable; riverine dilution and episodic low Ca^{2+}	High and stable marine alkalinity	[62,65]

Notes: Citation numbers follow the manuscript's reference list. Mechanistic links between salinity/Ca^{2+} and calcification or osmoregulation are indicated by [62,65].
